# The prevalence of contraceptive use among postpartum women and its associated factors during the early phase of COVID-19 outbreak: a time series study

**DOI:** 10.1186/s12978-024-01803-3

**Published:** 2024-06-06

**Authors:** Sarochinee Sathitloetsakun, Phanupong Phutrakool, Duangporn Maitreechit, Somsook Santibenchakul, Unnop Jaisamrarn, Pimpitcha Puangsricharoen

**Affiliations:** 1 Department of Obstetrics and Gynecology, Faculty of Medicine, Chulalongkorn University, King Chulalongkorn Memorial Hospital, Rama IV Road, Pathum Wan, Bangkok, 10330 Thailand; 2 Chula Data Management Center, Faculty of Medicine, Chulalongkorn University, Bangkok, 10330 Thailand; 3 Nursing Department, King Chulalongkorn Memorial Hospital, Bangkok, 10330 Thailand; 4 Department of Obstetrics and Gynecology, King Chulalongkorn Memorial Hospital, Bangkok, 10330 Thailand; 5 Division of Academic Affairs Faculty of Medicine, Chulalongkorn University, Bangkok, 10330 Thailand

**Keywords:** COVID-19, Thailand, Postpartum contraception, Long acting reversible contraception, Postpartum check-up

## Abstract

**Background:**

Unintended pregnancies can adversely affect maternal health, preventable through timely postpartum contraception. During the COVID-19 pandemic, family planning services were constrained by policies that curtailed outpatient visits. We investigated the prevalence of postpartum contraceptive initiation at King Chulalongkorn Memorial Hospital (KCMH) during January to June 2020, comparing with the same period in 2019, and identified factors associated with such initiation.

**Methods:**

We reviewed the medical records of 4506 postpartum women who delivered at KCMH during the study period. Logistic regression was conducted to test the association between early COVID-19 phase deliveries and post-partum long acting reversible contraception (LARC) initiation including copper intrauterine devices, levonorgestrel intrauterine systems, contraceptive implants, and progestogen-only injectable contraceptives.

**Results:**

A total of 3765 women (83.6%), of whom 1821 delivered during the pandemic and 1944 during the historical cohort period, were included in this study. The proportion of women who initiated non-permanent modern contraceptives at six weeks postpartum was comparable between the COVID-19 (73.4%) and historical cohort (75.3%) (*p* = 0.27) periods. The proportion of women who initiated LARC at six weeks postpartumwas comparable between the historical cohort period (22.5%) and the COVID-19 (19.7%) (*p* = 0.05) period. Accessing a six-week postpartum check-up was independently associated with LARC initiation, of which the adjusted odds ratio (OR) (95% confidence interval) was 3.01 (2.26 to 4.02).

**Conclusions:**

Our findings demonstrated that accessing postpartum care significantly associate with the use of LARC. The data suggest the strong influence of postpartum check-ups in facilitating the adoption of effective contraception, emphasizing the need for accessible postpartum care to sustain maternal health during health crises.

## Plain language summary

In this study, we looked at how often new mothers used birth control methods after giving birth during the COVID-19 pandemic in Thailand. We also wanted to find the factors that influenced their decision to use birth control. We reviewed the medical records of 4,506 women who had recently given birth at King Chulalongkorn Memorial Hospital. Out of these, 3,765 women were included in the study. Some gave birth during the pandemic, and others gave birth before the pandemic. We found that a similar number of women started using birth control about six weeks after giving birth, whether it was during the pandemic or before it. However, slightly fewer women chose very effective methods of birth control during the pandemic compared to before it. One important factor we found was that women who went for a check-up six weeks after giving birth were more likely to use highly effective birth control. This means that getting regular check-ups after childbirth can help women make better choices about birth control. In summary, our study showed that receiving postpartum care is closely linked to using LARC. This underlines how important it is for new mothers to have their check-ups after giving birth, to help them start using effective birth control if they choose.

## Introduction

Global health crises have profound impacts on healthcare systems by disrupting access to essential services including sexual and reproductive healthcare [1]. These disruptions are particularly significant during the postpartum period, a time when high motivation for contraceptive initiation was reported [2, 3]. The early phase of the COVID-19 pandemic saw an unprecedented a strain on healthcare resources, leading to a significant reduction in non-urgent care like postpartum follow-up. This was a critical time for postpartum women, who often rely on these appointments to initiate contraception [4]. This can lead to an increase in unintended and closely spaced pregnancies, which can negatively impact maternal physical and mental health [5–7]. The situation in Thailand during the COVID-19 pandemic mirrors the global trends, with significant increases in calls to the unintended pregnancy hotline, reflecting the challenges in accessing family planning services during the pandemic [8].

The importance of timely contraceptive initiation is underscored by the World Health Organization (WHO)’s recommendation for a two-year interval between pregnancies, as shorter intervals may pose adverse effects on maternal health [9]. The National Institute for Health and Care Excellence (NICE) guidelines categorize long-acting reversible contraception (LARCs) as critical during this period for their effectiveness and low discontinuation rates. This category includes copper intrauterine devices (IUDs), levonorgestrel intrauterine systems (LNG-IUS), contraceptive implants, and progestogen-only injectable contraceptives, all of which necessitate intervention by a healthcare provider for initiation [10, 11].

Initiating LARCs during the immediate postpartum period, before hospital discharge, can mitigate the barriers to accessing postpartum care, as women are already in a medical facility [12]. Despite the safety and efficacy of immediate postpartum contraception and recommended by medical society, in addition to female sterilization this approach has not gained much popularity in Thailand [12–14]. Thus, this study was conducted to determine the prevalence of immediate postpartum contraceptive initiation and during postpartum check-up amidst the COVID-19 pandemic in Thailand. We also sought to identify the factors associated with LARC initiation during this period. The findings are crucial in ensuring uninterrupted access to contraceptive services during pandemics and other healthcare system challenges. This may inform the policymakers and program implementers on how to maintain essential reproductive health services even in times of crisis.

## Methods

### Study design and population

This time series study was approved by the Institutional Review Board of the Faculty of Medicine, Chulalongkorn University (IRB No. 794/63). The study was conducted in accordance with the Declaration of Helsinki. We reviewed the electronic medical records of 4506 postpartum women who delivered at KCMH during the COVID-19 period (January 1 to June 30, 2020) and the historical cohort period (January 1 to June 30, 2019). January was selected as the initiation time point because, in Thailand, the first cases of COVID-19 were officially reported in this month [15]. Historical control of the same period was used because we aimed to mitigate the seasonality trend [16]. We excluded those who delivered before 22 weeks, underwent cesarean hysterectomy, or could not be contacted by telephone. To confirm the accuracy of data entry, electronic medical records were reviewed by two investigators (LS and SS). For women who did not return for a six-week postpartum visit at KCMH, an experienced family planning nurse conducted a five-minute telephonic interview. We repeated the phone call a maximum of five times. Informed consent was obtained from all eligible women before conducting the telephone interview.

### Measurements

Study data were collected and managed using REDCap electronic data capture tools hosted at KCMH [17]. The demographic information included age, ethnicity, marital status, address, and reimbursement. Obstetric characteristics included gravidity, number of living children, number of antenatal care (ANC) visits, pre-pregnancy body mass index (BMI), underlying medical conditions, obstetric complications, route of delivery, gestational age at delivery, birth weight, and APGAR score at 1 and 5 min. Data regarding immediate postpartum contraceptive initiation were gathered from the discharge summary, while data regarding six-week postpartum contraceptive initiation, among those who returned for postpartum visits at KCMH, were obtained from the electronic medical records of the family planning and reproductive health clinic. We confirmed the type of contraceptive initiation based on the prescription history. The primary outcome variable, contraceptive method use at six weeks postpartum, was defined as method initiation. The following methods are considered non-permanent modern contraceptives: combined oral contraceptive pills (COCs), combined hormonal contraceptive patch, depot medroxyprogesterone acetate (DMPA), progestin-only pills (POPs), contraceptive implant(s), copper IUD, LNG-IUS, and male condoms [18]. We interviewed participants to determine whether they were using any of the following in addition to the aforementioned methods: withdrawal method, lactation amenorrhea, or fertility awareness. For those who were using more than one method simultaneously, contraceptives that provided better efficacy were considered at the participants’ discretion [18]. Immediate postpartum contraceptive initiation was defined as contraceptive initiation either after delivery or before discharge from the postpartum hospital stay [12]. LARC was defined according to NICE guidelines [10].

### Study setting

At KCMH, we provide comprehensive contraceptive counseling to all postpartum women during the immediate postpartum period. Our facility offers an extensive range of immediate postpartum contraception options, including POPs, DMPA, contraceptive implant, IUDs. During the COVID-19 period, this approach has been emphasized. All medically eligible contraceptives were initiated upon request before hospital discharge. At the six-week postpartum visit, all women underwent a standardized contraceptive assessment by family planning nurses. All participants were educated with up-to-date evidence-based and precise information on each contraceptive method, its effectiveness, the risk of side effects, and tips on adherence. The women then selected their contraceptive method, which was provided by the family planning facility. During the historical cohort period, six-week postpartum visits at KCMH were scheduled for all women. However, during the COVID-19 period, only those with obstetric complications or medical diseases were scheduled to return for postpartum visits to KCMH. Others were asked to visit a nearby clinic or hospital for postpartum visits. In Thailand, COCs, POPs, and combined hormonal contraceptive patches are available over the counter, which women can access without a prescription.

### Statistical analysis

Statistical analyses were performed using SPSS version 22 (IBM Corp., Armonk, NY, USA) and STATA version 17 (StataCorp. 2021. Stata Statistical Software: Release 17. College Station, TX: StataCorp LLC.). Quantitative variables with a normal distribution were characterized using mean values and standard deviations (SDs), whereas those with a non-normal distribution were presented using medians and interquartile ranges (IQR). Qualitative variables were characterized by the number and percentage of participants in each category. Fisher’s exact test was used to test the association between the qualitative variables. Univariable logistic regression was conducted to test the association between delivery during the early phase of COVID-19 and the six-week post-partum LARC initiation. A backward logistic regression model was used to control for the potential confounders. The results were presented as odds ratios (OR) and 95% confidence intervals (CI). We tested the interaction between the COVID-19 period and follow-up at a family planning clinic to identify the association between these two variables and LARC initiation. The multicollinearity of the final model was tested using the variance inflation factor (VIF).

## Results

### Population

**Fig. 1:**
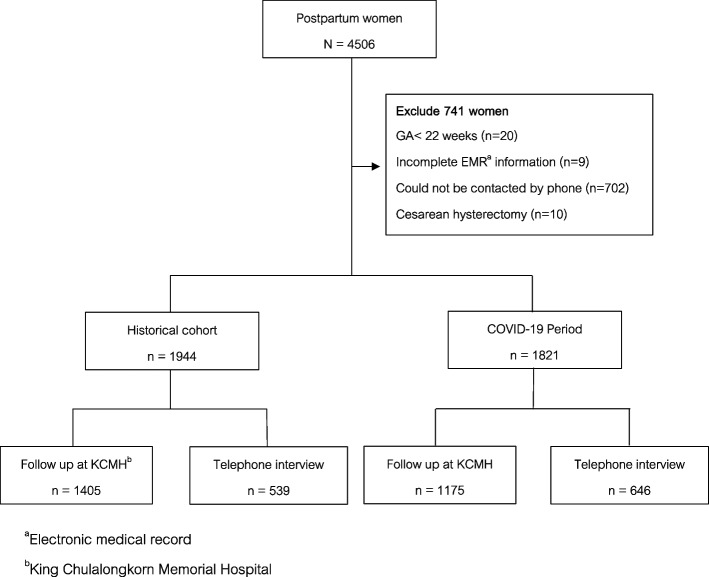
Study flow ^**a**^Electronic medical record. ^**b**^ King Chulalongkorn Memorial Hospital

A total of 3765 among 4506 women (83.6%), of whom 1821 delivered during the COVID-19 pandemic and 1944 during the historical cohort period, were included in this study (Fig. 1). The sociodemographic characteristics of the participants are presented in Table 1. Mean (SD) age between the COVID-19 and the historical cohort groups were comparable at 31.7 (5.5) and 31.0 (5.9) years, respectively. Almost all women (96.9%) were Thai. Most of them (69.8%) were married. The majority (46.7%) were primiparas after delivery of the most recent pregnancy. Approximately 35% of pre-pregnancy BMIs were in the range of overweight/obesity [19], of which the mean (SD) total weight gain was 13.3 (5.0) kg. Mean (SD) of total weight gain between the COVID-19 and the historical cohort groups were comparable at 13.2 (4.8) and 13.5 (5.1) kg, respectively. Around 5% developed preeclampsia during recent gestation, and approximately 9% had gestational diabetes. Approximately half delivered by cesarean section, and the majority (70%) delivered term newborns.

**Table 1:** Socio-demographic and obstetric characteristics of postpartum women at the King Chulalongkorn Memorial Hospital during COVID-19 (*N* = 3765)

Variable	COVID-19 period (*n* = 1821)	Historical cohort (*n* = 1944)	Total (*N* = 3765)	*p*-value*
	*n* (%)	*n* (%)	*N* (%)	
**Age (years)**				0.001
≤ 19	26 (1.43)	62 (3.19)	88 (2.34)	
> 19–24	186 (10.21)	217 (11.16)	403 (10.7)	
> 24–35	1110 (60.96)	1195 (61.47)	2305 (61.22)	
> 35–45	496 (27.24)	468 (24.07)	964 (25.6)	
> 45	3 (0.16)	2 (0.1)	5 (0.13)	
**Ethnicity**				0.160
Thai	1756 (96.43)	1891 (97.27)	3647 (96.87)	
Others^a^	65 (3.57)	53 (2.73)	118 (3.13)	
**Marital status**				0.255
Single	499 (27.4)	496 (25.51)	995 (26.43)	
Married	1258 (69.08)	1368 (70.37)	2626 (69.75)	
Divorce	1 (0.05)	5 (0.26)	6 (0.16)	
Widow	0	1 (0.05)	1 (0.03)	
No data of marital status	63 (3.46)	74 (3.81)	137 (3.64)	
**Residence**				0.232
Bangkok	1164 (63.92)	1279 (65.79)	2443 (64.89)	
Others	657 (36.08)	665 (34.21)	1322 (35.11)	
**Reimbursement**				0.001
Universal coverage	311 (17.08)	397 (20.42)	708 (18.8)	
Social security scheme	1171 (64.31)	1264 (65.02)	2435 (64.67)	
Self-paid	103 (5.66)	92 (4.73)	195 (5.18)	
KCMH^b^	74 (4.06)	47 (2.42)	121 (3.21)	
Government	160 (8.79)	144 (7.41)	304 (8.07)	
No data of reimbursement	2 (0.11)	0	2 (0.05)	
**Gravidity**				0.936
1	908 (46.71)	850 (46.68)	908 (46.71)	
2	647 (33.28)	620 (34.05)	647 (33.28)	
3	265 (13.63)	246 (13.51)	265 (13.63)	
4	94 (4.84)	78 (4.28)	94 (4.84)	
≥ 5	30 (1.54)	27 (1.48)	30 (1.54)	
**Living child** ^**c**^				0.163
0	1054 (57.88)	1181 (60.75)	2235 (59.36)	
1	596 (32.73)	593 (30.5)	1189 (31.58)	
2	142 (7.8)	131 (6.74)	273 (7.25)	
≥ 3	29 (1.59)	39 (2.01)	68 (1.81)	
**Number of ANC**				0.468
0	10 (0.55)	9 (0.46)	19 (0.5)	
1–4	64 (3.51)	83 (4.27)	147 (3.9)	
≥ 5	1747 (95.94)	1852 (95.27)	3599 (95.59)	
**Pre-Pregnancy body mass index (kg/m** ^**2**^ **)** [20]				0.713
Underweight < 18.5	287 (15.76)	316 (16.26)	603 (16.02)	
Normal 18.5-<23	853 (46.84)	935 (48.1)	1788 (47.49)	
Overweight 23-27.5	465 (25.54)	479 (24.64)	944 (25.07)	
Obesity > 27.5	216 (11.86)	213 (10.96)	429 (11.39)	
No data of pre-pregnancy BMI	0	1 (0.05)	1 (0.03)	
**Underlying medical disease**				
Yes	198 (10.87)	221 (11.37)	419 (11.13)	
No	1623 (89.13)	1723 (88.63)	3346 (88.87)	
***Hematologic disease*** ^***d***^				0.061
Yes	93 (5.11)	128 (6.58)	221 (5.87)	
No	1728 (94.89)	1816 (93.42)	3544 (94.13)	
***Endocrine disease*** ^***e***^				0.745
Yes	44 (2.42)	43 (2.21)	87 (2.31)	
No	1777 (97.58)	1901 (97.79)	3678 (97.69)	
***Psychiatric disease*** ^***f***^				0.043
Yes	12 (0.66)	4 (0.21)	16 (0.42)	
No	1809 (99.34)	1940 (99.79)	3749 (99.58)	
***Neurologic disease*** ^***g***^				> 0.999
Yes	8 (0.44)	9 (0.46)	17 (0.45)	
No	1813 (99.56)	1935 (99.54)	3748 (99.55)	
***Rheumatologic disease*** ^***h***^				0.296
Yes	14 (0.77)	9 (0.46)	23 (0.61)	
No	1807 (99.23)	1935 (99.54)	3742 (99.39)	
***Cancer*** ^***i***^				0.442
Yes	9 (0.49)	6 (0.31)	15 (0.40)	
No	1812 (99.51)	1938 (99.69)	3750 (99.60)	
**HIV status**				0.312
Positive	9 (0.49)	15 (0.77)	24 (0.64)	
Negative	1812 (99.51)	1929 (99.23)	3741 (99.36)	
**Hypertensive disorder**				0.624
No hypertensive disorder	1651 (90.66)	1784 (91.77)	3435 (91.24)	
Chronic hypertension	21 (1.15)	16 (0.82)	37 (0.98)	
Gestational hypertension	47 (2.58)	39 (2.01)	86 (2.28)	
Preeclampsia or eclampsia	89 (4.89)	92 (4.73)	181 (4.81)	
Chronic hypertension with superimpose preeclampsia	13 (0.71)	13 (0.67)	26 (0.69)	
**Diabetes**				0.130
No gestational diabetes	1649 (90.55)	1733 (89.15)	3382 (89.83)	
Gestational diabetes type 1	86 (4.72)	89 (4.58)	175 (4.65)	
Gestational diabetes type 2	70 (3.84)	91 (4.68)	161 (4.28)	
Overt DM	16 (0.88)	31 (1.59)	47 (1.25)	
**Placenta previa/low lying**				0.304
Yes	33 (1.81)	45 (2.31)	78 (2.07)	
No	1788 (98.19)	1899 (97.69)	3687 (97.93)	
**Previous cesarean section**				0.286
Yes	336 (18.45)	332 (17.08)	668 (17.74)	
No	1485 (81.55)	1612 (82.92)	3097 (82.26)	
**Previous myomectomy**				0.714
Yes	13 (0.71)	16 (0.82)	29 (0.77)	
No	1808 (99.29)	1928 (99.18)	3736 (99.23)	
**Multiple pregnancy**				0.300
Yes	86 (4.72)	78 (4.01)	164 (4.36)	
No	1735 (95.28)	1866 (95.99)	3601 (95.64)	
**Fetal complication** ^**j**^				0.048
Yes	61 (3.35)	44 (2.26)	105 (2.79)	
No	1760 (96.65)	1900 (97.74)	3660 (97.21)	
**Oligohydramnios or polyhydramnios**				0.241
Yes	34 (1.87)	26 (1.34)	60 (1.59)	
No	1787 (98.13)	1918 (98.66)	3705 (98.41)	
**Primary infertility**				0.195
Yes	85 (4.67)	74 (3.81)	159 (4.22)	
No	1736 (95.33)	1870 (96.19)	3606 (95.78)	
**Route of delivery**				0.192
Vaginal delivery	850 (46.68)	949 (48.82)	1799 (47.78)	
Cesarean section	971 (53.32)	995 (51.18)	1966 (52.22)	
**Gestational age at delivery**				0.818
22–28	27 (1.48)	29 (1.49)	56 (1.49)	
> 28–34	94 (5.16)	96 (4.94)	190 (5.05)	
> 34–37	446 (24.49)	453 (23.3)	899 (23.88)	
> 37	1254 (68.86)	1366 (70.27)	2620 (69.59)	
**Birthweight (grams)**				0.693
Very low birthweight (< 1500)	60 (3.29)	66 (3.4)	126 (3.35)	
Low birth weight (1500–2500)	223 (12.25)	215 (11.06)	438 (11.63)	
Normal birth weight (2500–4000)	1514 (83.14)	1634 (84.05)	3148 (83.61)	
Large for gestational age (> 4000)	24 (1.32)	29 (1.49)	53 (1.41)	
**APGAR score at 1 min**				0.101
0–3	19 (1.04)	31 (1.59)	50 (1.33)	
4–6	51 (2.8)	45 (2.31)	96 (2.55)	
7–10	1748 (95.99)	1868 (96.09)	3616 (96.04)	
No data of APGAR score at 1 minute^k^	3 (0.16)	0	3 (0.08)	

^*^Chi-square or Fisher’s exact tests as appropriate

^a^Others including Laos, Myanmar, and Gine

^b^King Chulalongkorn Memorial hospital

^c^Not including current pregnancy

^d^Including anemia, autoimmune hemolytic anemia, polycytemia, and thrombocytopenia

^e^Including hyperthyroid, hypothyroid, thyroid nodule, and autoimmune thyroiditis

^f^Including depression, bipolar, anxiety disorder, and panic disorder

^g^ Including stroke, epilepsy, myastinia gravis, multiple sclerosis, and migraine

^h^Including rheumatoid arthritis and systemic lupus erythematous

^i^Including thyroid cancer, breast cancer, colonic cancer, and lymphoma

^j^Including hydrops fetalis, fetal malposition, cleft lip and cleft palate, fetal growth restriction, large for gestational age, nonreassuring fetal status, and congenital heart disease

^k^Including patient whose birth occurred before hospital arrival

### Outcome data

During the COVID-19 period, the prevalence of immediate contraceptive use was 379/1821 (20.9%), and in the historical cohort period, 368/1944 (18.9%), (*p* = 0.05). Postpartum female sterilization was the most commonly selected method, with 17.0% and 17.9% in the COVID-19 and historical periods, respectively. The use of contraceptive implants was comparable between the COVID-19 (2.1%) and historical cohort periods (2.3%), as shown in Table 2. Excluding those who underwent postpartum female sterilization, the proportion of women who initiated non-permanent modern contraceptives at six weeks postpartum was comparable between the COVID-19 (73.4%, 1094/1490) and historical cohort periods (75.3%, 1217/1618) (*p* = 0.27), as shown in Table 3. The most commonly initiated method was male condoms in the COVID-19 (23.5%, 350/1490) and historical cohort periods (23.0%, 372/1618), followed by COCs in the COVID-19 (20.7%, 308/1490) and historical cohort periods (22.2%, 359/1618). The proportion of women who did not use contraception was comparable (22.6% and 21.9% during the COVID-19 and historical periods, respectively). The proportion of women who initiated LARC, were comparable between the historical cohort period (22.5%, 364/1618) and the COVID-19 period (19.7%, 293/1490) (*p* = 0.05).

**Table 2:** Type of immediate postpartum contraception initiation during the COVID-19 period (*n* = 3765)

Variables	COVID-19 period (*n* = 1821)	Historical cohort (*n* = 1944)	*p*-value*
	*n* (%)	*n* (%)	
Female sterilization	331 (17.03)	326 (17.90)	0.24
Contraceptive implant	40 (2.06)	41 (2.25)	
DMPA^a^	6 (0.31)	1 (0.05)	
Copper IUD^b^	2 (0.10)	0	
No contraception	1565 (80.50)	1453 (79.79)	

^*^Fisher’s exact test

^a^Depot medroxyprogesterone acetate

^b^Copper intrauterine device

**Table 3:** Contraception at six-weeks postpartum clinic excluding women who underwent postpartum female sterilization during the COVID-19 period (*n* = 3108)

Contraceptive method		COVID-19 period (*n* = 1490)		Historical cohort (*n* = 1618)	
		*n* (%)		*n* (%)	
Combined oral contraceptive pills		308 (20.67)		359 (22.19)	
DMPA^a^		162 (10.87)		212 (13.10)	
Progesterone only pills		143 (9.60)		122 (7.54)	
Contraceptive Implant		124 (8.32)		149 (9.21)	
Copper IUD^b^		4 (0.27)		3 (0.19)	
LNG-IUS^c^		3 (0.20)		0	
Male condoms		350 (23.49)		372 (22.99)	
Withdrawal		48 (3.22)		26 (1.61)	
Lactational amenorrhea		1 (0.07)		0	
Fertility awareness method		10 (0.67)		21 (1.30)	
No contraception		337 (22.62)		354 (21.88)	

^a^Depot medroxyprogesterone acetate

^b^Copper intrauterine device

^c^Levonorgestrel intrauterine device

Univariable analysis showed that the COVID-19 period tended to be associated with lower odds of initiating LARC compared to the historical cohort period, in which the OR (95% CI) was 0.83 (0.70−1.01), as shown in Table 4. This association was attenuated and did not reach statistical significance in the multivariable model, in which the adjusted OR (95% CI) was 0.92 (0.76−1.11). Postpartum follow-up at the family planning clinic was associated with higher odds of initiating LARC in the univariable model, in which the OR (95% CI) was 3.58 (2.82−4.54). This association was not attenuated in the multivariable model, in which the adjusted OR (95% CI) was 3.94 (2.96−5.23). We performed an additional analysis, including six-week postpartum visits outside our institution. The univariable analysis showed a smaller effect size, in which the OR (95% CI) was 2.48 (1.95−3.15). This association was not attenuated in the multivariate model, in which the adjusted OR (95% CI) was 3.01 (2.26−4.02). We tested the interaction between the COVID-19 and historical cohort periods and the six-week postpartum visit with LARC initiation but did not find a significant effect. Other sociodemographic and obstetric characteristics associated with higher odds of initiating LARC in the multivariable model were age < 35 years, having more than one child, having never attended antenatal care, and having delivered vaginally. These potential confounders were used to adjust the final multivariable model to test the association between the COVID-19 and historical cohort periods and six-week postpartum follow-up visits with LARC initiation. The VIF tests did not show multicollinearity among the adjusted variables in the final model.

**Table 4:** The association between the COVID-19 period and long acting reversible contraceptive initiation at six weeks postpartum

Variable	Univariable analysis		Multivariable analysis model 1^a^		Multivariable analysis model 2^b^	
	Odds ratio (95%CI)	*p*-value	Adjusted odds ratio (95% CI)	*p*-value	Adjusted odds ratio (95% CI)	*p*-value
**Exposure**						
Historical cohort	Reference	0.05	Reference	0.390	Reference	0.371
COVID-19 period	0.83 (0.70 to 1.01)		0.92 (0.76 to 1.11)		0.92 (0.76 to 1.11)	
**Age (year)**						
< 24	6.35 (4.73 to 8.52)	< 0.001	4.73 (3.33 to 6.73)	< 0.001	4.67 (3.28 to 6.65)	< 0.001
24–35	1.81 (1.43 to 2.29)		1.69 (1.30 to 2.21)		1.68 (1.28 to 2.19)	
> 35	Reference		Reference		Reference	
**Ethnicity**						
Thai	Reference	0.920				
Others^c^	1.02 (0.63 to 1.66)					
**Marital status**						
Single	1.12 (0.93 to 1.36)	0.122				
Married	Reference					
Others^d^	0.69 (0.42 to 1.14)					
**Residence**						
Bangkok	Reference	0.158	Reference	0.273	Reference	0.242
Others	0.88 (0.74 to 1.05)		0.89 (0.73 to 1.09)		0.89 (0.73 to 1.08)	
**Reimbursement**						
Universal coverage	1.63 (1.33 to 2.00)	< 0.001	1.15 (0.90 to 1.47)	0.252	1.16 (0.91 to 1.47)	0.238
Social security scheme	Reference		Reference		Reference	
Others^e^	0.94 (0.73 to 1.20)		0.96 (0.73 to 1.25)		0.96 (0.74 to 1.26)	
**Gravidity**						
1	Reference	0.166				
2	0.83 (0.69 to 1.01)					
≥ 3	0.95 (0.76 to 1.19)					
**Living child**						
0	Reference	0.083	Reference	0.017	Reference	0.044
1	0.93 (0.77 to 1.13)		1.30 (1.05 to 1.61)		1.25 (1.01 to 1.55)	
≥ 2	1.32 (1.00 to 1.75)		1.68 (1.21 to 2.34)		1.60 (1.15 to 2.23)	
**Number of ANC**						
0	5.39 (2.18 to 13.32)	< 0.001	4.67 (1.46 to 14.93)	0.009	5.10 (1.54 to 16.86)	0.008
1–4	1.35 (0.90 to 2.02)		1.21 (0.74 to 2.00)		1.22 (0.74 to 2.02)	
≥ 5	Reference		Reference		Reference	
**Pre-pregnancy body mass index (kg/m** ^**2**^ **)** [20]						
Underweight	1.29 (1.02 to 1.63)	0.217				
Normal	Reference					
Overweight	1.09 (0.89 to 1.35)					
Obesity	1.06 (0.80 to 1.40)					
**HIV status**						
HIV positive	1.58 (0.63 to 4.00)	0.352				
HIV negative	Reference					
**Hypertensive disorder**						
No	Reference	0.352				
Yes^f^	0.81 (0.59 to 1.11)					
**Diabetes**						
No	Reference	0.044	Reference	0.687	Reference	0.701
Yes^g^	0.74 (0.55 to 1.00)		0.93 (0.67 to 1.31)		0.94 (0.67 to 1.31)	
**Obstetrics risks**						
No	Reference	< 0.001				
Yes^h^	0.43 (0.36 to 0.51)					
**Route of delivery**						
Vaginal delivery	2.57 (2.16 to 3.07)	< 0.001	1.96 (1.60 to 2.39)	< 0.001	1.97 (1.61 to 2.40)	< 0.001
Cesarean section	Reference		Reference		Reference	
**Gestational age (week)**						
< 37	0.80 (0.66 to 0.97)	0.019	0.91 (0.73 to 1.13)	0.405	0.92 (0.74 to 1.14)	0.427
≥ 37	Reference		Reference		Reference	
**Birthweight (gram)**						
< 2500	0.71 (0.55 to 0.92)	0.012				
2500–4000	Reference					
> 4000	0.57 (0.24 to 1.35)					
**Follow up at KCMH** ^**i**^ **family planning clinic**						
Yes	3.58 (2.82 to 4.54)	< 0.001	3.94 (2.96 to 5.23)	< 0.001		
No	Reference		Reference			
**Follow up at family planning clinic**						
Yes	2.48 (1.95 to 3.15)	< 0.001			3.01 (2.26 to 4.02)	< 0.001
No	Reference				Reference	

^a^Multivariable analysis model 1: adjusted for age, address, reimbursement, living child, number of ANC, diabetes, route of delivery, gestational age, follow up at KCMH family planning clinic

^b^Multivariable analysis model 2: adjusted for age, address, reimbursement, living child, number of ANC, diabetes, route of delivery, gestational age, follow up at family planning clinic

^c^Others including Laos, Myanmar, and Gine

^d^Others including divorce and widow

^e^others including private insurance, self-paid, King Chulalongkorn Memorial Hospital officers, and government officers

^f^Including chronic hypertension, chronic hypertension with superimpose preeclampsia, gestational hypertension, and preeclampsia

^g^Including gestational diabetes type 1, gestational diabetes type 2, and overt diabetes

^h^Including placenta previa or low lying, Previous cesarean section, previous myomectomy, multiple pregnancy, fetal complication, oligohydramnios or polyhydramnios, Primary infertile, and other pregnancy risks

^i^King Chulalongkorn Memorial Hospital

## Discussion

The prevalence of immediate postpartum contraceptive initiation at the KCMH was comparable between the COVID-19 and the historical cohort periods. In addition to postpartum female sterilization, very few women initiated contraceptive use despite the recommendation of the medical societies about the efficacy and safety of these methods [12]. In our setting, the comprehensive contraceptive counseling is routinely provided in the postpartum ward and most women are eligible for the government’s LARC reimbursement benefits. This contrast points to the existence of barriers beyond the upfront payments for LARC. Further exploration into the clinician-patient discussion could provide additional insights on this issue. The lack of emphasis or detailed discussion by healthcare providers on the importance, efficacy, and safety of immediate postpartum contraceptive methods may be a significant barrier [20, 21]. The potential reasons for this gap in communication may include time constraints during consultation, clinicians’ perceptions or biases towards certain contraceptives, or possibly a lack of updated training regarding postpartum contraceptive options.

The six-week postpartum non-permanent modern contraceptive prevalence was comparable between the COVID-19 and historical cohort period, which differs from other settings [22–24]. This finding is notable because it contrasts with publications from other settings where the use of modern contraceptives decreased globally during the COVID-19 crisis, including in Asian countries [22–25]. Our study highlights a unique aspect of Thailand, where COCs, the preferred choice for contraception here, can be bought without a prescription [26]. The variation in study settings may explain the differences in our results compared to others.

Multivariable logistic regression analysis showed that the opportunity to return for postpartum visits influenced the use of LARC. Postpartum visits allows women to initiate LARC, a cost-saving strategy for preventing unintended pregnancy [27]. During the pandemic, in resource-limited settings, the demand for healthcare access increased, affecting non-urgent care, such as postpartum visits [28, 29]. LARC initiation immediately after giving birth is another strategy to ensure the prevention of the rising rate of unintended pregnancy during the pandemic [30].

The sociodemographic factor associated with LARC initiation was age. The increased odds of LARC use by younger women of reproductive age (< 24 years) may reflect the efforts to prioritize adolescents in pregnancy prevention programs in Thailand. According to the Act for Prevention and Solution of the Adolescent Pregnancy Problem, B.E. 2559, the government launched a policy to increase access by providing free LARC to 10 to 20-year-old women. Additionally, LARC can be initiated without a guardian’s consent [31]. This policy underscores the emphasis on reducing adolescent pregnancies by increasing contraceptive access among this age group. Older adolescents and young adults, particularly those in their early twenties, face different challenges and influences regarding contraceptive use. Being a university student or transitioning into higher education or the workforce can greatly impact their contraceptive needs, influencing behavior differently than younger adolescents targeted by current policies. The association between age and the unmet need for contraception is complex and depends on the demographic and societal context. For example, a study conducted among Guineans found a higher prevalence of unmet need for contraceptives among adolescents compared to young women as young women are more likely to live in union and may desire to have children while adolescents may face stigma of using contraceptive services outside their marriage [32]. Recognizing and addressing the unique needs of these age groups can enhance the effectiveness of reproductive health policies and better support individual’s needs.

An increasing number of children was associated with higher odds of LARC initiation. This association is consistent with that reported by Branum et al. [33]. Women with higher parity are usually more motivated to prevent further pregnancies. Postpartum women who had never come for antenatal care visits had higher odds of using LARC. This reflects our institute’s policy of providing special care to vulnerable groups at risk of further unintended pregnancy. Postpartum women who delivered vaginally had higher odds of using LARC than those who had cesarean sections. This discrepancy may be explained due to the exclusion of individuals who underwent postpartum tubal sterilization, a procedure commonly performed alongside cesarean deliveries [14].

The strengths of this analysis include analyzing a large dataset, considering numerous potential confounders for postpartum contraceptive use, and employing logistic regression to adjust for these confounders. Sensitivity and seasonal variation analyses ensured the reliability of the data regarding contraceptive use, particularly the initiation LARCs, which was verified through electronic medical records and prescription data. Nonprescription method assessments and telephonic interviews were conducted by an experienced family planning nurse. Another strength is that the KCMH is a tertiary care facility that can provide almost every modern contraceptive and provide same-visit contraceptive initiation for all requested methods. This contributes to an unbiased estimate owing to the availability of the methods.

However, this study had several limitations, including its quantitative nature that overlooks the qualitative reasons behind contraceptive choices and the lack of consideration for partners’ opinions. The reliance on electronic medical records, which may not be comprehensive, could introduce bias, although this is mitigated by cross-referencing with prescription records. The study’s single-center design and focus on the early COVID-19 phase in Thailand limit its generalizability and relevance to other pandemic peaks. Further research should focus on contraceptive prevalence during each peak of the pandemic periods, as well as the efficacy of employing telemedicine as an adjunctive tool to encourage postpartum contraceptive initiation, as this has been widely used in Thailand since the peak of the pandemic [34]. Furthermore, investigating the interval between postpartum delivery and follow-up visits for initiating contraception presents another valuable research avenue. This exploration could shed light on how this timing affects postpartum mothers’ contraceptive choices and outcomes.

## Conclusion

Our findings demonstrated the six-week postpartum visit was a significant factor in initiating LARC, providing an opportunity for women to access clinician-initiated contraceptive methods. The findings underscore the vital importance of postpartum check-ups in enabling the uptake of effective contraception, highlighting the urgent need for accessible postpartum care to ensure the continuation of maternal health services during health crises.

## Data Availability

The data that support the findings of this study are available from the corresponding author (SS2) upon reasonable request.

## Abbreviations

KCMH: King Chulalongkorn Memorial Hospital
NICE: National Institute for Health and Care Excellence
Copper IUD: copper intrauterine devices
LNG-IUS: levonorgestrel intrauterine system
LARC: long-acting reversible contraceptive
ANC: antenatal care
BMI: body mass index
COCs: combined oral contraceptive pills
DMPA: depot medroxyprogesterone acetate
POPs: progestin-only pills
SDs: standard deviations
OR: odds ratios
CI: confidence intervals
VIF: variance inflation factor
